# Inhibition of PI3K/AKT and MAPK/ERK pathways causes activation of FOXO transcription factor, leading to cell cycle arrest and apoptosis in pancreatic cancer

**DOI:** 10.1186/1750-2187-5-10

**Published:** 2010-07-19

**Authors:** Sanjit K Roy, Rakesh K Srivastava, Sharmila Shankar

**Affiliations:** 1 Department of Pharmacology, Toxicology and Therapeutics, and Medicine, The University of Kansas Cancer Center, The University of Kansas Medical Center, 3901 Rainbow Boulevard, Kansas City, KS, 66160, USA; 2 Department of Pathology and Laboratory Medicine, The University of Kansas Cancer Center, The University of Kansas Medical Center, 3901 Rainbow Boulevard, Kansas City, KS, 66160, USA

## Abstract

**Background:**

Mammalian forkhead members of the class O (FOXO) transcription factors, including FOXO1, FOXO3a, and FOXO4, are implicated in the regulation of several biological processes, including the stress resistance, metabolism, cell cycle, apoptosis and DNA repair. The objectives of this study were to examine the molecular mechanisms by which FOXO transcription factors induced cell cycle arrest and apoptosis and enhanced anti-proliferative effects of sulforaphane (SFN, an active compound in cruciferous vegetables) in pancreatic cancer cells.

**Results:**

Our data demonstrated that SFN inhibited cell proliferation and colony formation, and induced apoptosis through caspase-3 activation in pancreatic cancer cells. The inhibition of PI3K/AKT and MEK/ERK pathways activated FOXO transcription factors. SFN inhibited phosphorylation of AKT and ERK, and activated FOXO transcription factors, leading to cell cycle arrest and apoptosis. Phosphorylation deficient mutants of FOXO proteins enhanced FOXO transcriptional activity, and further enhanced SFN-induced FOXO activity and apoptosis. SFN induced the expression of p21^/CIP1 ^and p27^/KIP1^, and inhibited the expression of cyclin D1.

**Conclusion:**

These data suggest that inhibition of PI3K/AKT and ERK pathways acts together to activate FOXO transcription factor and enhances SFN-induced FOXO transcriptional activity, leading to cell cycle arrest and apoptosis.

## Background

Cancer of the pancreas is the fourth leading cause of cancer death in the United States. This year approximately 32,000 Americans will die from cancer of the pancreas. With an overall 5-year survival rate of 3% [1], pancreatic cancer has one of the poorest prognoses among all cancers [2]. Only 20% of pancreatic cancer patients are eligible for surgical resection, which currently remains the only potentially curative therapy [3]. Unfortunately, many cancers of the pancreas are not resectable at the time of diagnosis. There are limited treatment options available for this disease because chemo- and radio-therapies are largely ineffective, and metastatic disease frequently redevelops even after surgery [1,2]. Therefore, developing effective strategies to prevent pancreatic neoplasms are of paramount importance.

Sulforaphane (SFN), a constituent of cruciferous vegetables, is a naturally occurring isothiocyanate with promising chemopreventive activity [4]. Epidemiological studies have shown that people who eat cruciferous vegetables have reduced incidence of breast and prostate cancer. SFN possesses anti-oxidant, anti-proliferative and anti-carcinogenic properties [5-7]. SFN is effective in preventing chemically induced breast [8,9], stomach [5] and colon [10] cancers in rats. We and others have shown that SFN inhibited the growth of prostate, breast, oral and squamous carcinoma xenografts [11-15]. SFN enhanced radiosensitivity of tumor cells *in vitro *and *in vivo *[16]. Furthermore, a pharmacokinetic study has demonstrated that it is rapidly absorbed and 82% bioavailable [17]. SFN induces a phase 2 enzyme, thereby neutralizing carcinogens before they can damage DNA [18,19]. SFN inhibits benzo[a]pyrene-DNA and 1,6-dinitropyrene-DNA adducts formation [20-23], and downregulates PI3K/AKT [24,25] and NFκB [12,26,27] pathways. We have recently demonstrated that SFN induces death receptors (DR4 and DR5) and proapoptotic members of Bcl-2 family, inhibits antiapoptotic Bcl-2 proteins, activates caspase(s), and enhances apoptosis-inducing potential of TRAIL in vitro [12]. *In vivo*, SFN inhibits growth of PC-3 cells orthotopically implanted in nude mice by inducing apoptosis and inhibiting tumor cell proliferation, metastasis and angiogenesis [12]. These studies strongly suggest that SFN can be developed as a cancer preventive agent.

PTEN (phosphatase and tensin homolog deleted on chromosome 10, also called MMAC1 or TEP1) is a tumor suppressor gene [28-30], which is frequently deleted or mutated in a wide range of human cancers, including glioblastoma [31], melanoma [32], and prostate [33], breast [34], and endometrial cancers [35]. While point mutations in PTEN rarely occur in pancreatic cancer [36,37], functional inactivation of PTEN through promoter methylation [38], loss of protein expression [39], reduction of mRNA levels [40], or loss of heterozygocity (LOH) of linked markers [37,41] occur with high frequency. Phosphatidylinositol 3,4,5-trisphosphate (PIP_3_) is a substrate of PTEN [42-44]. AKT is a serine-threonine protein kinase regulated by PIP_3 _that is implicated in survival signaling in a wide a variety of cells, including fibroblastic, epithelial, and neuronal cells [45]. PTEN increases sensitivity to cell death in response to several apoptotic stimuli by negatively regulating the PI3K/AKT pathway [43]. In addition to its role in regulating the PI3K/AKT cell survival pathway, PTEN also inhibits growth factor-induced Shc phosphorylation and suppresses the MAP kinase signaling pathway [46], suggesting that PTEN has roles in independent of PI3K/AKT signaling pathway. Hyperactivation of AKT is associated with resistance to apoptosis, increased cell growth, cell proliferation, metastasis, angiogenesis, and cellular energy metabolism [45,47-54]. Overexpression of AKT has been reported in a variety of human cancers, including pancreatic cancer, and cells expressing elevated levels of AKT are less sensitive to apoptosis stimuli [38,55-57]. Antagonizing PI3K activity negatively regulates AKT activity. Once activated, however, AKT exerts antiapoptotic effects through phosphorylation of substrates such as Bad [58,59] and caspase-9 [60] that directly regulate the apoptotic machinery, or human telomerase reverse transcriptase subunit [61], forkhead transcription family members [62,63] and IB kinases [64] that indirectly inhibit apoptosis [65]. Studies in pancreatic cancer cell lines have demonstrated that PI3K is required for growth and survival of tumor cells [66-68]. Furthermore, amplification or activation of AKT2 occurs in up to 60% of pancreatic cancer [39,69-71], supporting the participation of an activated PI3K-AKT axis in this disease.

FOXO subfamily of forkhead transcription factors include FOXO1a/FKHR, FOXO3a/FKHRL1, and FOXO4/AFX [72-75]. The PI3K pathway, via activation of its downstream kinase AKT, phosphorylates each of the FOXO proteins [62,76,77]. These phosphorylations result in impairment of DNA binding ability and increased binding affinity for the 14-3-3 protein [62,77]. Newly formed 14-3-3-FOXO complexes are then exported from the nucleus [78], thereby inhibiting FOXO-dependent transcription. Inhibition of the PI3K pathway leads to dephosphorylation and nuclear translocation of active FKHRL1, FKHR, and AFX; which induce cells cycle arrest and apoptosis [79]. Conversely, loss of PTEN activity results in increased AKT activity leading to inhibition of FOXO protein activity through phosphorylation and cytoplasmic sequestration. In addition, the data demonstrate that FOXO transcriptional activity controls cellular proliferation and apoptosis downstream of PTEN [80,81]. FOXO regulates cell cycle and apoptotic genes such as cyclin-dependent kinase inhibitor (CKI) p27^KIP1 ^[78,80,82,83], Bim [84,85], Fas ligand [62], and Bcl-6 [86]. Consequently, activation of the PI3K pathway serves to repress FOXO-mediated growth arrest and apoptosis. However, regulation of FOXO target genes is multifactorial, and therefore other transcription factors and post-translation regulatory events will influence the final level of protein expression. Interestingly, overexpression of AKT, and inactivation and loss of PTEN are frequently observed in pancreatic cancer [39,66-71], indicating a potential role for FOXOs in modulating both cell cycle and apoptosis during tumorigenesis and treatment. Together, these results indicate that FOXO proteins are important downstream effectors of PTEN tumor suppressive activity; however, their molecular targets and mechanisms of action in pancreatic cancer are not well understood.

The Ras proteins are small (21 kDa) GTP-binding, membrane-associated proteins [87]. The Ras proteins transduce signals from ligand-activated tyrosine kinase receptors to downstream effectors [88]. Activating mutations can impair GTP hydrolysis and lead to constitutively activated Ras that impacts the cellular phenotype [89]. Oncogenic Ras can lead to cellular transformation [90], presumably by perturbing its signal transduction pathways. Ras regulates multiple signaling pathways [91]. Three major groups of MAP kinases are found in mammalian cells: extracellular signal-regulated protein kinase (ERK) [92], p38 MAP kinase [93], and c-Jun N-terminal kinase (JNK) [94-96]. MAP kinases regulate many cellular activities, which range from gene expression to mitosis, movement, metabolism, and apoptosis. These MAP kinases are activated by the dual phosphorylations of neighboring threonine and tyrosine residues in response to various extracellular stimuli [97,98]. Specifically, p38 and JNK have been implicated in stress-responsive signaling leading to the initiation of adaptive events such as gene expression, differentiation, metabolism, and apoptosis [94,95,99]. ERKs are often activated by growth signals, such as epidermal growth factor (EGF) or platelet-derived growth factor [100]. We have recently demonstrated that inhibition of PI3K/AKT and MEK/ERK pathways act synergistically to regulate antiangiogenic effects of EGCG and SFN through activation of FOXO transcription factors [24,101].

Furthermore, FOXO transcription factors play a crucial role in the regulation of tissue homeostasis in organs such as the pancreas, and complex diseases such as diabetes and cancer. Unfortunately, the intracellular mechanisms by which SFN inhibits growth and induces apoptosis in pancreatic cancer cells through regulation of FOXO transcription factors have never been examined. The objectives of our study were to examine the molecular mechanisms by which FOXO transcription factors induce cell cycle arrest and apoptosis and enhances the anti-proliferative effects of SFN in pancreatic cancer cells. Our results demonstrate that inhibition of PI3K/AKT and ERK pathways activates FOXO transcription factors. SFN inhibited phosphorylation of AKT and ERK, and dephosphorylated FOXO transcription factors, leading to cell cycle arrest and apoptosis. Phosphorylation deficient mutants of FOXO proteins enhanced FOXO transcriptional activity, and further enhanced SFN-induced FOXO activity.

## Results

### Sulforaphane (SFN) inhibits cell growth in human pancreatic cancer cells

**Figure 1:**
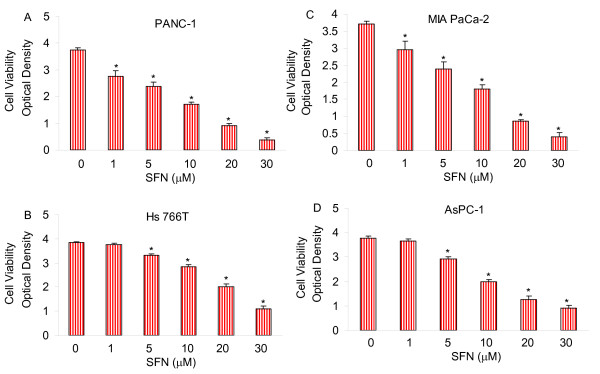
**Effect of sulforaphane (SFN) on viability of pancreatic cancer cells**. Pancreatic cancer (PANC-1, MIA PaCa-2, Hs766T and AsPC-1) cells were treated with SFN (0-30 μM) for 48 h. Cell viability was measured by XTT assay. Data represent the mean ± S.D. * = significantly different from respective controls, P < 0.05.

We first examined the effects of SFN on cell proliferation in four pancreatic cancer cell lines by XTT assay. We have selected four pancreatic cancer cell lines (MIA PaCa-2, AsPC-1, PANC-1 and Hs766T) because they have been derived from different pathological stages and may thus respond differently to SFN [102,103]. MIA PaCa-2 harbors a point mutation on Kras gene resulting in amino acid sunbstitution from the wild-type glycine to a valine at codon 12. AsPC-1 and PANC-1 harbor a point mutation on Kras gene resulting in amino acid substitution from glycine to aspartate. Hs766T cell line does not possess a point mutation in codon 12 of the Kras gene. SFN inhibited cell viability in a dose dependent manner (Fig. 1). PANC-1 and MIA PaCa-2 cell lines were most sensitive, AsPC-1 cell line was moderately sensitive, and Hs 766T cell line was least sensitive. These data suggest that SFN can be a viable agent for inhibiting pancreatic cancer cell proliferation.

### Sulforaphane inhibits colony formation in human pancreatic cancer cells

**Figure 2:**
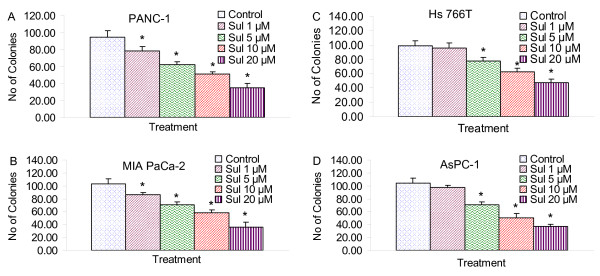
**Effect of sulforaphane (SFN) on colony formation**. Pancreatic cancer (PANC-1, MIA PaCa-2, Hs766T and AsPC-1) cells were treated with SFN (0-20 μM), and number of colonies were counted. Data represent the mean ± S.D. * = significantly different from respective controls, P < 0.05.

We next examined the effects of SFN on colony formation (a characteristic of cancer) on four pancreatic cancer cell lines by soft agar assay. SFN inhibited colony formation in a dose dependent manner (Fig. 2). Colonies formed by PANC-1 and MIA PaCa-2 cells were most sensitive, AsPC-1 cell line was moderately sensitive, and Hs 766T cell line was least sensitive. These data suggest that SFN can be used as a potent chemopreventive agent for pancreatic cancer.

### Sulforaphane induces caspase-3 activation in human pancreatic cancer cell

**Figure 3:**
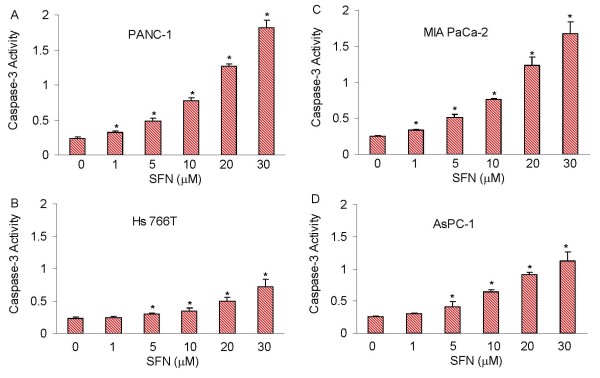
**Effect of sulforaphane (SFN) on caspase-3 activity**. Pancreatic cancer PANC-1, MIA PaCa-2, Hs 766T and AsPC-1 cells were treated with SFN (0-30 μM) for 12 h and caspase-3 activity was measured as per manufacturer's instructions (EMD Biosciences). Data represent the mean ± S.D. * = significantly different from respective controls, P < 0.05.

Most chemopreventive agents induce apoptosis through mitochondrial pathway, which activates caspase-3 [104]. We therefore examined whether SFN-induced apoptosis through caspase-3 activation in pancreatic cancer cell lines (Fig. 3). SFN induced caspase-3 activity in PANC-1, MIA PaCa-2, Hs 766T and AsPC-1 cells. However, a relatively high dose of SFN was required to activate caspase-3 in Hs 766T cells compared to other pancreatic cancer cell lines. These data suggest that SFN induced apoptosis through caspase-3 activation and may engage the mitochondria.

### Regulation and function of PI3K/AKT and MAP kinase pathways by sulforaphane

**Figure 4:**
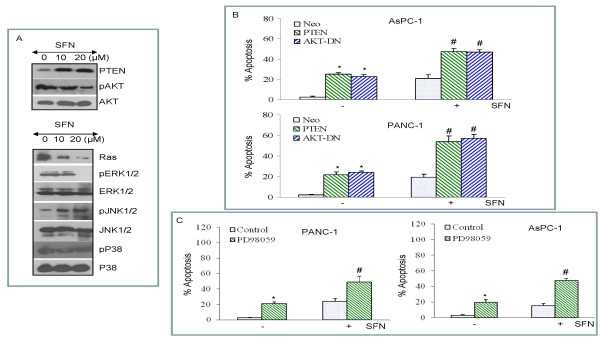
**Effects of sulforaphane (SFN) on the expression of PTEN, AKT, and MAP kinases; and the effects of PI3K/AKT and MAPK pathways on SFN-induced apoptosis**. (A), PANC-1 cells were treated with or without SFN (0-20 μM) for 24 h. The cells were harvested and the expression of PTEN, phospho-AKT, AKT, Ras, phospho-ERK, ERK, phospho-JNK, JNK, phospho-p38 and p38 was measured by Western blotting. (B), PTEN and dominant negative AKT enhance SFN-induced apoptosis. AsPC-1 and PANC-1 cells were transiently transfected with empty vector (pcDNA3.1), PTEN wild type (PTEN-WT) or dominant negative AKT (AKT-DN) along with pCMV-LacZ vector (as transfection control) for 24 h. After medium replacement, cells were treated with SFN (10 μM) for 48 h and, apoptosis was measured by Live Dead Assay. Data represent the mean ± S.D. *, # = significantly different from respective controls, P < 0.05. (C), MEK inhibitor PD98059 enhances SFN-induced apoptosis. AsPC-1 and PANC-1 cells were pretreated with PD98059 (1 μM) followed by treatment with SFN (10 μM) for 48 h and, apoptosis was measured by Live Dead Assay. Data represent the mean ± S.D. *, # = significantly different from respective controls, P < 0.05.

In most cancer cells, AKT is constitutively active and enhances cell proliferation [105]. In order to understand a relationship between PTEN and AKT in SFN-induced apoptosis, we measured the expression of PTEN and phosphorylation status of AKT in cells treated with SFN (Fig. 4A). SFN induces PTEN expression and inhibits AKT phosphorylation in pancreatic cancer PANC-1 cells. By comparison, SFN has no effect on total AKT expression. These data suggest that SFN inhibits cell proliferation by regulating PI3K/AKT pathway.

Ras/Raf/MAP kinase pathway regulates many cellular activities, which range from gene expression to mitosis, movement, metabolism, and apoptosis [94,106-109]. We therefore examined the effects of SFN on the expression of Ras, and activation of ERK, JNK and p38 MAP kinases. SFN inhibited Ras expression in PANC-1 cells (Fig. 4A). Treatment of PANC-1 cells with SFN caused a decrease in ERK phosphorylation, and an increase in JNK phosphorylation. SFN has no significant effect on p38 MAP kinase activity in PANC-1 cells. These data suggest that SFN inhibits growth and induces apoptosis through regulation of Ras/Raf/MAP kinase pathway.

We next examined whether SFN induces apoptosis through PI3K/AKT pathway (Fig. 4B). Pancreatic cancer cells were transfected with empty vector, wild type PTEN, dominant negative AKT (DN-AKT), and apoptosis was measured. Overexpression of wild type PTEN or DN-AKT induced apoptosis in AsPC-1 and PANC-1 cells. Treatment of transfected cells with SFN further enhanced apoptosis. These data suggest that inhibition of PI3K/AKT pathway enhances SFN-induced apoptosis in pancreatic cancer cells.

We next examined whether inhibition of MEK/ERK pathway enhances SFN-induced apoptosis in pancreatic cancer cells. MEK1/2 inhibitor (PD98059) induced apoptosis in PANC-1 and AsPC-1 cells (Fig. 4C). PD98059 enhanced SFN-induced apoptosis. Overall, these data suggest that inhibition of PI3K/AKT and MEK/ERK pathways enhanced SFN-induced apoptosis.

### Sulforaphane induces p21^/WAF1/CIP1^, and p27^/KIP1 ^and inhibits cyclin D1

**Figure 5:**
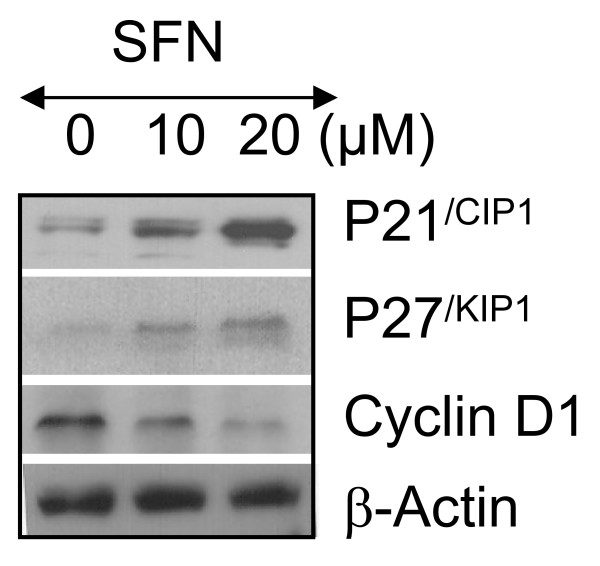
**Effects of sulforaphane (SFN) on cell cycle regulatory genes**. PANC-1 cells were treated with SFN (0-20 μM) for 24 h. The expression of p21^/CIP1^, p27^/KIP1 ^and cyclin D1 was measured by Western blotting. Anti β-actin antibody was used as a loading control.

PI3K/AKT signaling pathway may be involved in the control of the cell cycle progression most likely through mechanisms involving the activation of FOXO transcription factors [82]. We next examined the effects of SFN on cell cycle regulatory genes. SFN induced the expression cell cycle inhibitors p21^/WAF1/CIP1 ^and p27^/KIP1^, and inhibited the expression of cyclin D1 in PANC-1 cells (Fig. 5). These data suggest that SFN causes growth arrest by regulating expression of cell cycle genes.

### Overexpression of FOXO transcription factors inhibits cell viability and enhances FOXO transcriptional activity in pancreatic cancer cells

**Figure 6:**
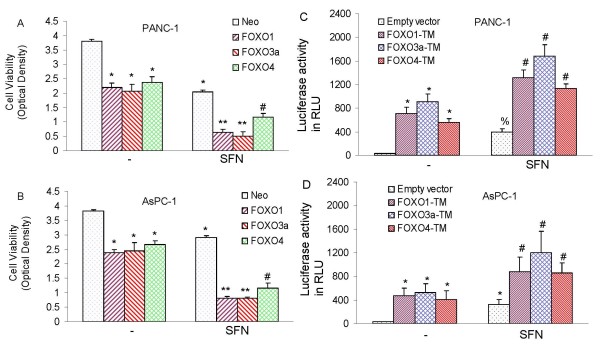
**Effects of FOXO transcription factors on cell viability and FOXO transcriptional activity**. (A and B), PANC-1 and AsPC-1 cells were transiently transfected with plasmids expressing neo (pcDNA3.1), FOXO1, FOXO3a, or FOXO4 along with pCMV-LacZ vector (as transfection control). After transfection, cells were treated with or without SFN (10 μM) for 48 h, and cell viability was measured by XTT assay. Data represent the mean ± S.D. * = significantly different from respective controls, P < 0.05. (C and D), Phosphorylation deficient mutants of FOXO enhance sulforaphane-induced FOXO transcriptional activity in pancreatic cancer. PANC-1 and AsPC-1 cells were transiently transfected with empty vector or constructs encoding FOXO1-TM, FOXO3a-TM, or FOXO4-TM together with 6X DBE-luciferase for 24 h. After transfection, cells were washed with RPMI, treated with SFN (10 μM) for 24 h, and harvested for firefly/Renilla luciferase assays using the Dual-Luciferase Reporter Assay System (Promega). Luciferase counts were normalized using *Renilla *luciferase transfection control (pRL-TK; Promega). Data represent the mean ± S.D. * = significantly different from respective controls, P < 0.05.

In order to examine whether FOXO transcription factors affect the ability of SFN to inhibit cell viability, pancreatic cancer cells were transfected with FOXO1, FOXO3a or FOXO4 (Fig. 6A and 6B). FOXO expression plasmids and FOXO-luciferase construct (pGL3-6X DBE) have previously been described [101]. Overexpression of FOXO1, FOXO3a, and FOXO4 inhibited cell viability in PANC-1 and AsPC-1 cells. The inhibitory effects of SFN on cell viability were further enhanced when pancreatic cancer cells were transfected with FOXO1, FOXO3a, and FOXO4. These data suggest that FOXO transcription factors can enhance the antiproliferative effects of SFN.

We next examined whether SFN induces transcriptional activation of FOXO in the presence or absence phosphorylation deficient triple mutants of FOXO proteins (FOXO1-TM, FOXO3a-TM, or FOXO4-TM). PANC-1 and AsPC-1 cells were transfected with wild type FOXO promoter linked to a luciferase reporter gene in the presence or absence of plasmids expressing FOXO1-TM, FOXO3a-TM, or FOXO4-TM (Fig. 6C and 6D). After transfection, cells were treated with SFN for 24 h, and luciferase activity was measured. Transfection of cells with plasmids expressing FOXO1-TM, FOXO3a-TM, or FOXO4-TM induced FOXO transcriptional activity compared with the empty vector (control). SFN-induced FOXO transcriptional activity was further enhanced in the presence of FOXO1-TM, FOXO3a-TM, and FOXO4-TM. These data indicate that FOXO transcription factor may play a major role in mediating biological effects of SFN in pancreatic cancer cells.

### Inhibition of PI3K/AKT and MEK/ERK pathways synergistically/additively induces FOXO transcriptional activity and apoptosis in the presence or absence of sulforaphane

**Figure 7:**
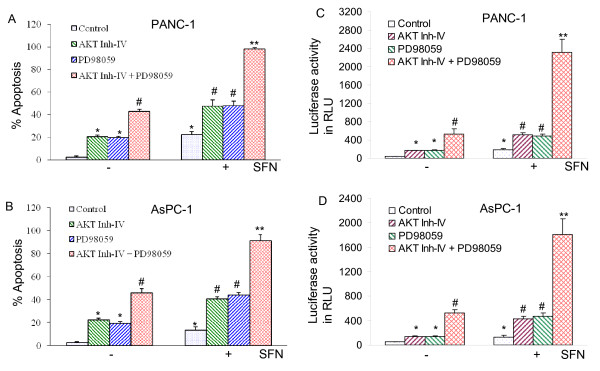
**Inhibition of PI3K/AKT and MEK/ERK pathways synergistically/additively enhanced sulforaphane (SFN)-induced apoptosis and FOXO transcriptional activity in pancreatic cancer cells**. (A and B), PANC-1 and AsPC-1 cells were pretreated with AKT inhibitor IV (1 μM) and/or MEK1/2 inhibitor PD98059 (10 μM) for 2 h, followed by treatment with SFN (10 μM) or DMSO (control) for 48 h. At the end of incubation period, cells were harvested and apoptosis was measured by TUNEL assay. Data represent mean ± SD. * = significantly different from respective controls, P < 0.05. (C and D), PANC-1 and AsPC-1 cells were transiently transfected with 6X DBE-luciferase construct for 24 h. After transfection, cells were pretreated with AKT inhibitor IV (1 μM) and/or MEK1/2 inhibitor PD98059 (10 μM) for 2 h, followed by treatment with SFN (10 μM) or DMSO (control) for 24 h. Cells were harvested for firefly/Renilla luciferase assays using the Dual-Luciferase Reporter Assay System (Promega). Luciferase counts were normalized using *Renilla *luciferase transfection control (pRL-TK; Promega). Data represent the mean ± S.D. *, #, ** = significantly different from respective controls, P < 0.05.

Since inhibition of PI3K/AKT and MEK/ERK pathways induce apoptosis in pancreatic cancer cells, we sought to examine whether these pathways act together to regulate SFN-induced apoptosis. AKT inhibitor (AKT Inh-IV) and MEK1/2 inhibitor (PD98059) synergistically/additively induced apoptosis in PANC-1 and AsPC-1 cells (Fig. 7A and 7B). AKT inhibitor and PD98059 alone enhanced SFN-induced apoptosis. Interestingly, the combination of AKT inhibitor and PD98059 with SFN induced more apoptosis than AKT inhibitor plus SFN or PD98059 plus SFN. These data suggest that inhibition of PI3K/AKT and MEK/ERK pathways act synergistically/additively to regulate apoptosis in the absence or presence of SFN.

Since inhibition of PI3K/AKT and MEK/ERK pathways synergistically/additively induces apoptosis in pancreatic cancer cells, we sought to examine whether inhibition of these two pathways act together to regulate FOXO activity. AKT inhibitor (AKT Inh-IV) and MEK1/2 inhibitor (PD98059) synergistically induced FOXO transcriptional activity in AsPC-1 and PANC-1 cells (Fig. 7C and 7D). AKT inhibitor or PD98059 enhanced SFN-induced FOXO transcriptional activity. Interestingly, the combination of AKT Inh-IV and PD98059 with SFN induced greater FOXO transcriptional activity than AKT Inh-IV plus SFN or PD98059 plus SFN. These data suggest that inhibition of PI3K/AKT and MEK/ERK pathways acts synergistically/additively to regulate FOXO transcriptional activity in the absence or presence of SFN.

## Discussion

Our study demonstrates, for the first time, that cancer preventive effects of SFN are regulated through activation of FOXO transcription factors. Specifically, we have demonstrated that (i) SFN induces apoptosis through caspase-3 activation, and causes growth arrest through induction of p21 and p27 and inhibition of cyclin D1; (ii) SFN induces apoptosis through inhibition of both PI3K/AKT and MEK/ERK pathways, and activation of FOXO transcription factors; (iii) inhibition of PI3K/AKT and MEK/ERK pathways acts together to enhance the activation of FOXO transcription factors; and (iv) phosphorylation deficient mutants of FOXO proteins further enhance SFN-induced FOXO activity and apoptosis. Our data are in agreement with others who demonstrated the anticancer activity of SFN in pancreatic cancer [110-112].

FOXO transcription factors play a crucial role in the regulation of tissue homeostasis in organs such as the pancreas and the ovaries and complex diseases such as diabetes and cancer [113-117]. FOXO transcription factors are emerging as critical transcriptional integrators among pathways regulating differentiation, proliferation, survival, and angiogenesis [118-121]. FOXO transcription factors regulate angiogenesis and postnatal neovascularization by regulation angiopoietin 2 (Ang2) and eNOS [121]. Gene expression profiling showed that FOXO1 and FOXO3a specifically regulate a nonredundant but overlapping set of angiogenesis- and vascular remodeling-related genes [121]. The FOXO1-deficient mice died around embryonic day 11 because of defects in the branchial arches and remarkably impaired vascular development of embryos and yolk sacs [118]. We have recently demonstrated that inhibition of the MEK/ERK and PI3K/AKT pathways synergistically induced FOXO transcriptional activity and inhibited angiogenesis (cell migration and capillary tube formation); these events were further enhanced in the presence of SFN [24]. Phosphorylation deficient mutants of FOXO enhanced antiangiogenic effects of SFN by activating the FOXO transcription factor. These studies suggest that activation of FOXO transcription factor by SFN could be an important physiological process to inhibit angiogenesis which may ultimately control tumor growth.

Activation of Kras has been shown to activate both PI3K/AKT and MAPK pathways [24,101,122-124]. Oxidative stress and activation of the JNK pathway induce the nucleocytoplasmic translocation of the pancreatic transcription factor Pdx-1, which leads to pancreatic β-cell dysfunction [125,126]. Furthermore, FOXO1/FKHR plays a role as a mediator between the JNK pathway and Pdx-1 [127]. Under oxidative stress conditions, FOXO1 changed its intracellular localization from the cytoplasm to the nucleus in the pancreatic β-cell line HIT-T15. The overexpression of JNK also induced the nuclear localization of FOXO1, but in contrast, suppression of JNK reduced the oxidative stress-induced nuclear localization of FOXO1, suggesting the involvement of the JNK pathway in FOXO1 translocation. In addition, oxidative stress or activation of the JNK pathway decreased the activity of AKT in HIT cells, leading to the decreased phosphorylation of FOXO1 following nuclear localization. Furthermore, adenovirus-mediated FOXO1 overexpression reduced the nuclear expression of Pdx-1, whereas repression of FOXO1 by FOXO1-specific small interfering RNA retained the nuclear expression of Pdx-1 under oxidative stress conditions. Activation of ERK has been shown to phosphorylate FOXO proteins, resulting in nuclear exclusion and transcriptional repression. In addition to ERK, direct phosphorylation of FOXO by AKT results in cytoplasmic retention and inactivation, inhibiting the expression of FOXO-regulated genes, which control the cell cycle, cell death, cell metabolism and oxidative stress [82,128,129]. Taken together, these studies demonstrate that dephosphorylation and activation of FOXO by inhibition of PI3K/AKT and MEK/ERK pathways has significant implication for pancreatic cancer treatment and prevention, where Kras is activated in about 90% patients.

In addition to phosphorylation, the acetylation/deacetylation of FOXO can be regulated by p300, Cbp (CREB-binding protein) and Pcaf (p300/CBP-associated factors) in response to oxidative stress or DNA binding, followed by deacetylation by class I and II histone deacetylases [130-132], including Sirt1, the NAD^+^-dependent deacetylase encoded by the ortholog of yeast longevity gene Sir2 [133]. Therefore, further studies are needed to examine the consequences of acetylation/deacetylation of FOXO transcription factors on anti-proliferative and anti-angiogenic effects of SFN.

In conclusion, we have demonstrated that SFN induces cell cycle arrest and apoptosis through regulation of FOXO transcription factors. Pharmacological and genetic inhibitions of PI3K/AKT and MEK/ERK pathways can have synergistic effects on the activation of FOXO transcription factors through dephosphorylation and nuclear retention. Thus, SFN appears to be as an attractive agent for pancreatic cancer prevention and treatment.

## Methods

### Reagents

Antibodies against PTEN, phospho-AKT, AKT, phospho-ERK, ERK, phospho-p38, p38, p21/CIP1, p27/KIP1, cyclin D1, and β-actin were purchased from Cell Signaling Technology, Inc. (Danvers, MA). Enhanced chemiluminescence (ECL) Western blot detection reagents were from Amersham Life Sciences Inc. (Arlington Heights, IL). Terminal Deoxynucleotidyl Transferase Biotin-dUTP Nick End Labeling (TUNEL) assay kit was purchased from EMD Biosciences/Calbiochem (San Diego, CA). Sulforaphane was purchased from LKT Laboratories, Inc. (St. Paul, MN). Kits for Terminal Deoxynucleotidyl Transferase Biotin-dUTP Nick End Labeling (TUNEL) and caspase-3 assays were purchased from EMD Biosciences/Calbiochem (San Diego, CA).

### Cell Culture

PANC-1, MIA PaCa-2, AsPC-1 and Hs 766T cells were obtained from the American Type Culture Collection (Manassas, VA) and cultured in RPMI 1640 supplemented with 10% fetal bovine serum (FBS) and 1% antibiotic-antimycotic (Invitrogen) at 37°C in a humidified atmosphere of 95% air and 5% CO_2_^.^

### Western Blot Analysis

Western blots were performed as we described earlier [134,135]. In brief, cells were lysed in RIPA buffer containing 1 × protease inhibitor cocktail, and protein concentrations were determined using the Bradford assay (Bio-Rad, Philadelphia, PA). Proteins were separated by 12.5% SDS/PAGE and transferred to membranes (Millipore, Bedford, MA) in a Tris (20 mM), glycine (150 mM) and methanol (20%) buffer at 55 V for 4 h at 4°C. After blocking in 5% nonfat dry milk in TBS, the membranes were incubated with primary antibodies at 1:1,000 dilution in TBS overnight at 4°C, washed three times with TBS-Tween 20, and then incubated with secondary antibodies conjugated with horseradish peroxidase at 1:5,000 dilution in TBS for 1 hour at room temperature. Membranes were washed again in TBS-Tween 20 for three times at room temperature. Protein bands were visualized on X-ray film using an enhanced chemiluminescence detection system.

### Caspase-3 Assay

Cells (3 × 10^4 ^per well) were seeded in a 96-well plate with 200 μl culture medium. Approximately 16 h later, cells were treated with various doses of SFN to induce apoptosis. Casapse-3 activity was measured by a fluorometer as per manufacturer's instructions (EMD Biosciences).

### Statistical Analysis

The mean and SD were calculated for each experimental group. Differences between groups were analyzed by one or two way ANOVA, followed by Bonferoni's multiple comparison tests using PRISM statistical analysis software (GrafPad Software, Inc., San Diego, CA). Significant differences among groups were calculated at P < 0.05.

## List of abbreviations used

ANOVA: Analysis of Variance; PTEN: Phosphatase and Tensin Homolog Deleted on Chromosome 10; RIPA: Radio-Immunoprecipitation Assay; SDS-PAGE: Sodium Dodecyl Sulfate-Polyacrylamide Gel Electrophoresis; SFN: Sulforaphane; TBS: Tris Buffer Saline.

## Competing interests

The authors declare that they have no competing interests.

## Authors' contributions

SKR and SS performed the experiments. SS and RKS designed and wrote the manuscript. All the authors have read and approved the final manuscript.

## References

[B1] Warshaw AL Fernandez-del Castillo C Pancreatic carcinoma N Engl J Med 1992 326 455 465 1732772 10.1056/NEJM199202133260706

[B2] Magee CJ Ghaneh P Neoptolemos JP Surgical and medical therapy for pancreatic carcinoma Best Pract Res Clin Gastroenterol 2002 16 435 455 10.1053/bega.2002.0317 12079268

[B3] Yeo TP Hruban RH Leach SD Wilentz RE Sohn TA Kern SE Iacobuzio-Donahue CA Maitra A Goggins M Canto MI Pancreatic cancer Curr Probl Cancer 2002 26 176 275 10.1067/mcn.2002.129579 12399802

[B4] Fahey JW Talalay P Antioxidant functions of sulforaphane: a potent inducer of Phase II detoxication enzymes Food Chem Toxicol 1999 37 973 979 10.1016/S0278-6915(99)00082-4 10541453

[B5] Fahey JW Haristoy X Dolan PM Kensler TW Scholtus I Stephenson KK Talalay P Lozniewski A Sulforaphane inhibits extracellular, intracellular, and antibiotic-resistant strains of Helicobacter pylori and prevents benzo[a]pyrene-induced stomach tumors Proc Natl Acad Sci USA 2002 99 7610 7615 10.1073/pnas.112203099 12032331 PMC124299

[B6] Park EJ Pezzuto JM Botanicals in cancer chemoprevention Cancer Metastasis Rev 2002 21 231 255 10.1023/A:1021254725842 12549763

[B7] Weisburger JH Antimutagens, anticarcinogens, and effective worldwide cancer prevention J Environ Pathol Toxicol Oncol 1999 18 85 93 15281219

[B8] Zhang Y Kensler TW Cho CG Posner GH Talalay P Anticarcinogenic activities of sulforaphane and structurally related synthetic norbornyl isothiocyanates Proc Natl Acad Sci USA 1994 91 3147 3150 10.1073/pnas.91.8.3147 8159717 PMC43532

[B9] Fahey JW Zhang Y Talalay P Broccoli sprouts: an exceptionally rich source of inducers of enzymes that protect against chemical carcinogens Proc Natl Acad Sci USA 1997 94 10367 10372 10.1073/pnas.94.19.10367 9294217 PMC23369

[B10] Chung FL Conaway CC Rao CV Reddy BS Chemoprevention of colonic aberrant crypt foci in Fischer rats by sulforaphane and phenethyl isothiocyanate Carcinogenesis 2000 21 2287 2291 10.1093/carcin/21.12.2287 11133820

[B11] Singh AV Xiao D Lew KL Dhir R Singh SV Sulforaphane induces caspase-mediated apoptosis in cultured PC-3 human prostate cancer cells and retards growth of PC-3 xenografts in vivo Carcinogenesis 2004 25 83 90 10.1093/carcin/bgg178 14514658

[B12] Shankar S Ganapathy S Srivastava RK Sulforaphane enhances the therapeutic potential of TRAIL in prostate cancer orthotopic model through regulation of apoptosis, metastasis and angiogenesis Clinical Cancer Res 2008 14 1 16 10.1158/1078-0432.CCR-08-0903 18980980

[B13] Jackson SJ Singletary KW Sulforaphane inhibits human MCF-7 mammary cancer cell mitotic progression and tubulin polymerization J Nutr 2004 134 2229 2236 15333709 10.1093/jn/134.9.2229

[B14] Kim JH Han Kwon K Jung JY Han HS Hyun Shim J Oh S Choi KH Choi ES Shin JA Leem DH Sulforaphane Increases Cyclin-Dependent Kinase Inhibitor, p21 Protein in Human Oral Carcinoma Cells and Nude Mouse Animal Model to Induce G(2)/M Cell Cycle Arrest J Clin Biochem Nutr 2010 46 60 67 20104266 10.3164/jcbn.09-65 PMC2803134

[B15] Cho NP Han HS Leem DH Choi IS Jung JY Kim HJ Moon KS Choi KH Soh Y Kong G Sulforaphane enhances caspase-dependent apoptosis through inhibition of cyclooxygenase-2 expression in human oral squamous carcinoma cells and nude mouse xenograft model Oral Oncol 2009 45 654 660 10.1016/j.oraloncology.2008.07.003 18805045

[B16] Yu D Sekine-Suzuki E Xue L Fujimori A Kubota N Okayasu R Chemopreventive agent sulforaphane enhances radiosensitivity in human tumor cells Int J Cancer 2009 125 1205 1211 10.1002/ijc.24480 19452523

[B17] Hanlon N Coldham N Gielbert A Kuhnert N Sauer MJ King LJ Ioannides C Absolute bioavailability and dose-dependent pharmacokinetic behaviour of dietary doses of the chemopreventive isothiocyanate sulforaphane in rat Br J Nutr 2008 99 559 564 10.1017/S0007114507824093 17868493

[B18] Rushmore TH Kong AN Pharmacogenomics, regulation and signaling pathways of phase I and II drug metabolizing enzymes Curr Drug Metab 2002 3 481 490 10.2174/1389200023337171 12369894

[B19] Misiewicz I Skupinska K Kowalska E Lubinski J Kasprzycka-Guttman T Sulforaphane-mediated induction of a phase 2 detoxifying enzyme NAD(P)H:quinone reductase and apoptosis in human lymphoblastoid cells Acta Biochim Pol 2004 51 711 721 15448733

[B20] Bacon JR Williamson G Garner RC Lappin G Langouet S Bao Y Sulforaphane and quercetin modulate PhIP-DNA adduct formation in human HepG2 cells and hepatocytes Carcinogenesis 2003 24 1903 1911 10.1093/carcin/bgg157 12949046

[B21] Kensler TW Chen JG Egner PA Fahey JW Jacobson LP Stephenson KK Ye L Coady JL Wang JB Wu Y Effects of glucosinolate-rich broccoli sprouts on urinary levels of aflatoxin-DNA adducts and phenanthrene tetraols in a randomized clinical trial in He Zuo township, Qidong, People's Republic of China Cancer Epidemiol Biomarkers Prev 2005 14 2605 2613 10.1158/1055-9965.EPI-05-0368 16284385

[B22] Conaway CC Wang CX Pittman B Yang YM Schwartz JE Tian D McIntee EJ Hecht SS Chung FL Phenethyl isothiocyanate and sulforaphane and their N-acetylcysteine conjugates inhibit malignant progression of lung adenomas induced by tobacco carcinogens in A/J mice Cancer Res 2005 65 8548 8557 10.1158/0008-5472.CAN-05-0237 16166336

[B23] Singletary K MacDonald C Inhibition of benzo[a]pyrene- and 1,6-dinitropyrene-DNA adduct formation in human mammary epithelial cells bydibenzoylmethane and sulforaphane Cancer Lett 2000 155 47 54 10.1016/S0304-3835(00)00412-2 10814878

[B24] Davis R Singh KP Kurzrock R Shankar S Sulforaphane inhibits angiogenesis through activation of FOXO transcription factors Oncol Rep 2009 22 1473 1478 19885601 10.3892/or_00000589

[B25] Jakubikova J Sedlak J Mithen R Bao Y Role of PI3K/Akt and MEK/ERK signaling pathways in sulforaphane- and erucin-induced phase II enzymes and MRP2 transcription, G2/M arrest and cell death in Caco-2 cells Biochem Pharmacol 2005 69 1543 1552 10.1016/j.bcp.2005.03.015 15896333

[B26] Cheung KL Kong AN Molecular targets of dietary phenethyl isothiocyanate and sulforaphane for cancer chemoprevention AAPS J 2010 12 87 97 10.1208/s12248-009-9162-8 20013083 PMC2811646

[B27] Choi S Lew KL Xiao H Herman-Antosiewicz A Xiao D Brown CK Singh SV D,L-Sulforaphane-induced cell death in human prostate cancer cells is regulated by inhibitor of apoptosis family proteins and Apaf-1 Carcinogenesis 2007 28 151 162 10.1093/carcin/bgl144 16920735

[B28] Li J Yen C Liaw D Podsypanina K Bose S Wang SI Puc J Miliaresis C Rodgers L McCombie R PTEN, a putative protein tyrosine phosphatase gene mutated in human brain, breast, and prostate cancer Science 1997 275 1943 1947 10.1126/science.275.5308.1943 9072974

[B29] Steck PA Pershouse MA Jasser SA Yung WK Lin H Ligon AH Langford LA Baumgard ML Hattier T Davis T Identification of a candidate tumour suppressor gene, MMAC1, at chromosome 10q23.3 that is mutated in multiple advanced cancers Nat Genet 1997 15 356 362 10.1038/ng0497-356 9090379

[B30] Li DM Sun H TEP1, encoded by a candidate tumor suppressor locus, is a novel protein tyrosine phosphatase regulated by transforming growth factor beta Cancer Res 1997 57 2124 2129 9187108

[B31] Wang SI Puc J Li J Bruce JN Cairns P Sidransky D Parsons R Somatic mutations of PTEN in glioblastoma multiforme Cancer Res 1997 57 4183 4186 9331071

[B32] Guldberg P thor Straten P Birck A Ahrenkiel V Kirkin AF Zeuthen J Disruption of the MMAC1/PTEN gene by deletion or mutation is a frequent event in malignant melanoma Cancer Res 1997 57 3660 3663 9288767

[B33] Cairns P Okami K Halachmi S Halachmi N Esteller M Herman JG Jen J Isaacs WB Bova GS Sidransky D Frequent inactivation of PTEN/MMAC1 in primary prostate cancer Cancer Res 1997 57 4997 5000 9371490

[B34] Rhei E Kang L Bogomolniy F Federici MG Borgen PI Boyd J Mutation analysis of the putative tumor suppressor gene PTEN/MMAC1 in primary breast carcinomas Cancer Res 1997 57 3657 3659 9288766

[B35] Tashiro H Blazes MS Wu R Cho KR Bose S Wang SI Li J Parsons R Ellenson LH Mutations in PTEN are frequent in endometrial carcinoma but rare in other common gynecological malignancies Cancer Res 1997 57 3935 3940 9307275

[B36] Sakurada A Suzuki A Sato M Yamakawa H Orikasa K Uyeno S Ono T Ohuchi N Fujimura S Horii A Infrequent genetic alterations of the PTEN/MMAC1 gene in Japanese patients with primary cancers of the breast, lung, pancreas, kidney, and ovary Jpn J Cancer Res 1997 88 1025 1028 9439675 10.1111/j.1349-7006.1997.tb00324.x PMC5921310

[B37] Okami K Wu L Riggins G Cairns P Goggins M Evron E Halachmi N Ahrendt SA Reed AL Hilgers W Analysis of PTEN/MMAC1 alterations in aerodigestive tract tumors Cancer Res 1998 58 509 511 9458098

[B38] Asano T Yao Y Zhu J Li D Abbruzzese JL Reddy SA The PI 3-kinase/Akt signaling pathway is activated due to aberrant Pten expression and targets transcription factors NF-kappaB and c-Myc in pancreatic cancer cells Oncogene 2004 23 8571 8580 10.1038/sj.onc.1207902 15467756

[B39] Altomare DA Tanno S De Rienzo A Klein-Szanto AJ Tanno S Skele KL Hoffman JP Testa JR Frequent activation of AKT2 kinase in human pancreatic carcinomas J Cell Biochem 2003 88 470 476 10.1002/jcb.10287 14735903

[B40] Ebert MP Fei G Schandl L Mawrin C Dietzmann K Herrera P Friess H Gress TM Malfertheiner P Reduced PTEN expression in the pancreas overexpressing transforming growth factor-beta 1 Br J Cancer 2002 86 257 262 10.1038/sj.bjc.6600031 11870516 PMC2375189

[B41] Hahn SA Seymour AB Hoque AT Schutte M da Costa LT Redston MS Caldas C Weinstein CL Fischer A Yeo CJ Allelotype of pancreatic adenocarcinoma using xenograft enrichment Cancer Res 1995 55 4670 4675 7553647

[B42] Maehama T Dixon JE The tumor suppressor, PTEN/MMAC1, dephosphorylates the lipid second messenger, phosphatidylinositol 3,4,5-trisphosphate J Biol Chem 1998 273 13375 13378 10.1074/jbc.273.22.13375 9593664

[B43] Stambolic V Suzuki A de la Pompa JL Brothers GM Mirtsos C Sasaki T Ruland J Penninger JM Siderovski DP Mak TW Negative regulation of PKB/Akt-dependent cell survival by the tumor suppressor PTEN Cell 1998 95 29 39 10.1016/S0092-8674(00)81780-8 9778245

[B44] Tamura M Gu J Matsumoto K Aota S Parsons R Yamada KM Inhibition of cell migration, spreading, and focal adhesions by tumor suppressor PTEN Science 1998 280 1614 1617 10.1126/science.280.5369.1614 9616126

[B45] Kulik G Weber MJ Akt-dependent and -independent survival signaling pathways utilized by insulin-like growth factor I Mol Cell Biol 1998 18 6711 6718 9774684 10.1128/mcb.18.11.6711 PMC109254

[B46] Gu J Tamura M Yamada KM Tumor suppressor PTEN inhibits integrin- and growth factor-mediated mitogen-activated protein (MAP) kinase signaling pathways J Cell Biol 1998 143 1375 1383 10.1083/jcb.143.5.1375 9832564 PMC2133067

[B47] Downward J PI 3-kinase, Akt and cell survival Semin Cell Dev Biol 2004 15 177 182 10.1016/j.semcdb.2004.01.002 15209377

[B48] Harrington LS Findlay GM Lamb RF Restraining PI3K: mTOR signalling goes back to the membrane Trends Biochem Sci 2005 30 35 42 10.1016/j.tibs.2004.11.003 15653324

[B49] Dudek H Datta SR Franke TF Birnbaum MJ Yao R Cooper GM Segal RA Kaplan DR Greenberg ME Regulation of neuronal survival by the serine-threonine protein kinase Akt Science 1997 275 661 665 10.1126/science.275.5300.661 9005851

[B50] Kaufmann SH Hengartner MO Programmed cell death: alive and well in the new millennium Trends Cell Biol 2001 11 526 534 10.1016/S0962-8924(01)02173-0 11719060

[B51] Kennedy SG Wagner AJ Conzen SD Jordan J Bellacosa A Tsichlis PN Hay N The PI 3-kinase/Akt signaling pathway delivers an anti-apoptotic signal Genes Dev 1997 11 701 713 10.1101/gad.11.6.701 9087425

[B52] Khwaja A Akt is more than just a Bad kinase Nature 1999 401 33 34 10.1038/43354 10485701

[B53] Chen X Thakkar H Tyan F Gim S Robinson H Lee C Pandey SK Nwokorie C Onwudiwe N Srivastava RK Constitutively active Akt is an important regulator of TRAIL sensitivity in prostate cancer Oncogene 2001 20 6073 6083 10.1038/sj.onc.1204736 11593415

[B54] Kandasamy K Srivastava RK Role of the phosphatidylinositol 3'-kinase/PTEN/Akt kinase pathway in tumor necrosis factor-related apoptosis-inducing ligand-induced apoptosis in non-small cell lung cancer cells Cancer Res 2002 62 4929 4937 12208743

[B55] Cicenas J Urban P Vuaroqueaux V Labuhn M Kung W Wight E Mayhew M Eppenberger U Eppenberger-Castori S Increased level of phosphorylated akt measured by chemiluminescence-linked immunosorbent assay is a predictor of poor prognosis in primary breast cancer overexpressing ErbB-2 Breast Cancer Res 2005 7 R394 401 10.1186/bcr1015 15987444 PMC1175052

[B56] Semba S Moriya T Kimura W Yamakawa M Phosphorylated Akt/PKB controls cell growth and apoptosis in intraductal papillary-mucinous tumor and invasive ductal adenocarcinoma of the pancreas Pancreas 2003 26 250 257 10.1097/00006676-200304000-00008 12657951

[B57] Yao Z Okabayashi Y Yutsudo Y Kitamura T Ogawa W Kasuga M Role of Akt in growth and survival of PANC-1 pancreatic cancer cells Pancreas 2002 24 42 46 10.1097/00006676-200201000-00006 11741181

[B58] Datta SR Dudek H Tao X Masters S Fu H Gotoh Y Greenberg ME Akt phosphorylation of BAD couples survival signals to the cell-intrinsic death machinery Cell 1997 91 231 241 10.1016/S0092-8674(00)80405-5 9346240

[B59] del Peso L Gonzalez-Garcia M Page C Herrera R Nunez G Interleukin-3-induced phosphorylation of BAD through the protein kinase Akt Science 1997 278 687 689 10.1126/science.278.5338.687 9381178

[B60] Cardone MH Roy N Stennicke HR Salvesen GS Franke TF Stanbridge E Frisch S Reed JC Regulation of cell death protease caspase-9 by phosphorylation Science 1998 282 1318 1321 10.1126/science.282.5392.1318 9812896

[B61] Kang SS Kwon T Kwon DY Do SI Akt protein kinase enhances human telomerase activity through phosphorylation of telomerase reverse transcriptase subunit J Biol Chem 1999 274 13085 13090 10.1074/jbc.274.19.13085 10224060

[B62] Brunet A Bonni A Zigmond MJ Lin MZ Juo P Hu LS Anderson MJ Arden KC Blenis J Greenberg ME Akt promotes cell survival by phosphorylating and inhibiting a Forkhead transcription factor Cell 1999 96 857 868 10.1016/S0092-8674(00)80595-4 10102273

[B63] Kops GJ de Ruiter ND De Vries-Smits AM Powell DR Bos JL Burgering BM Direct control of the Forkhead transcription factor AFX by protein kinase B Nature 1999 398 630 634 10.1038/19328 10217147

[B64] Romashkova JA Makarov SS NF-kappaB is a target of AKT in anti-apoptotic PDGF signalling Nature 1999 401 86 90 10.1038/43474 10485711

[B65] Ozes ON Mayo LD Gustin JA Pfeffer SR Pfeffer LM Donner DB NF-kappaB activation by tumour necrosis factor requires the Akt serine-threonine kinase Nature 1999 401 82 85 10.1038/43466 10485710

[B66] Perugini RA McDade TP Vittimberga FJ Jr Callery MP Pancreatic cancer cell proliferation is phosphatidylinositol 3-kinase dependent J Surg Res 2000 90 39 44 10.1006/jsre.2000.5833 10781373

[B67] Shah SA Potter MW Hedeshian MH Kim RD Chari RS Callery MP PI-3' kinase and NF-kappaB cross-signaling in human pancreatic cancer cells J Gastrointest Surg 2001 5 603 612 discussion 612-603 10.1016/S1091-255X(01)80102-5 12086898

[B68] Bondar VM Sweeney-Gotsch B Andreeff M Mills GB McConkey DJ Inhibition of the phosphatidylinositol 3'-kinase-AKT pathway induces apoptosis in pancreatic carcinoma cells in vitro and in vivo Mol Cancer Ther 2002 1 989 997 12481421

[B69] Cheng JQ Ruggeri B Klein WM Sonoda G Altomare DA Watson DK Testa JR Amplification of AKT2 in human pancreatic cells and inhibition of AKT2 expression and tumorigenicity by antisense RNA Proc Natl Acad Sci USA 1996 93 3636 3641 10.1073/pnas.93.8.3636 8622988 PMC39663

[B70] Ruggeri BA Huang L Wood M Cheng JQ Testa JR Amplification and overexpression of the AKT2 oncogene in a subset of human pancreatic ductal adenocarcinomas Mol Carcinog 1998 21 81 86 10.1002/(SICI)1098-2744(199802)21:2<81::AID-MC1>3.0.CO;2-R 9496907

[B71] Schlieman MG Fahy BN Ramsamooj R Beckett L Bold RJ Incidence, mechanism and prognostic value of activated AKT in pancreas cancer Br J Cancer 2003 89 2110 2115 10.1038/sj.bjc.6601396 14647146 PMC2376856

[B72] Galili N Davis RJ Fredericks WJ Mukhopadhyay S Rauscher FJ Emanuel BS Rovera G Barr FG Fusion of a fork head domain gene to PAX3 in the solid tumour alveolar rhabdomyosarcoma Nat Genet 1993 5 230 235 10.1038/ng1193-230 8275086

[B73] Anderson MJ Viars CS Czekay S Cavenee WK Arden KC Cloning and characterization of three human forkhead genes that comprise an FKHR-like gene subfamily Genomics 1998 47 187 199 10.1006/geno.1997.5122 9479491

[B74] Hillion J Le Coniat M Jonveaux P Berger R Bernard OA AF6q21, a novel partner of the MLL gene in t(6;11)(q21;q23), defines a forkhead transcriptional factor subfamily Blood 1997 90 3714 3719 9345057

[B75] Borkhardt A Repp R Haas OA Leis T Harbott J Kreuder J Hammermann J Henn T Lampert F Cloning and characterization of AFX, the gene that fuses to MLL in acute leukemias with a t(X;11)(q13;q23) Oncogene 1997 14 195 202 10.1038/sj.onc.1200814 9010221

[B76] Van Der Heide LP Hoekman MF Smidt MP The ins and outs of FoxO shuttling: mechanisms of FoxO translocation and transcriptional regulation Biochem J 2004 380 297 309 10.1042/BJ20040167 15005655 PMC1224192

[B77] Guo S Rena G Cichy S He X Cohen P Unterman T Phosphorylation of serine 256 by protein kinase B disrupts transactivation by FKHR and mediates effects of insulin on insulin-like growth factor-binding protein-1 promoter activity through a conserved insulin response sequence J Biol Chem 1999 274 17184 17192 10.1074/jbc.274.24.17184 10358076

[B78] Medema RH Kops GJ Bos JL Burgering BM AFX-like Forkhead transcription factors mediate cell-cycle regulation by Ras and PKB through p27kip1 Nature 2000 404 782 787 10.1038/35008115 10783894

[B79] Nakamura N Ramaswamy S Vazquez F Signoretti S Loda M Sellers WR Forkhead transcription factors are critical effectors of cell death and cell cycle arrest downstream of PTEN Mol Cell Biol 2000 20 8969 8982 10.1128/MCB.20.23.8969-8982.2000 11073996 PMC86551

[B80] Dijkers PF Medema RH Pals C Banerji L Thomas NS Lam EW Burgering BM Raaijmakers JA Lammers JW Koenderman L Coffer PJ Forkhead transcription factor FKHR-L1 modulates cytokine-dependent transcriptional regulation of p27(KIP1) Mol Cell Biol 2000 20 9138 9148 10.1128/MCB.20.24.9138-9148.2000 11094066 PMC102172

[B81] Dijkers PF Birkenkamp KU Lam EW Thomas NS Lammers JW Koenderman L Coffer PJ FKHR-L1 can act as a critical effector of cell death induced by cytokine withdrawal: protein kinase B-enhanced cell survival through maintenance of mitochondrial integrity J Cell Biol 2002 156 531 542 10.1083/jcb.200108084 11815629 PMC2173339

[B82] Cappellini A Tabellini G Zweyer M Bortul R Tazzari PL Billi AM Fala F Cocco L Martelli AM The phosphoinositide 3-kinase/Akt pathway regulates cell cycle progression of HL60 human leukemia cells through cytoplasmic relocalization of the cyclin-dependent kinase inhibitor p27(Kip1) and control of cyclin D1 expression Leukemia 2003 17 2157 2167 10.1038/sj.leu.2403111 12931221

[B83] Burgering BM Kops GJ Cell cycle and death control: long live Forkheads Trends Biochem Sci 2002 27 352 360 10.1016/S0968-0004(02)02113-8 12114024

[B84] Dijkers PF Medema RH Lammers JW Koenderman L Coffer PJ Expression of the pro-apoptotic Bcl-2 family member Bim is regulated by the forkhead transcription factor FKHR-L1 Curr Biol 2000 10 1201 1204 10.1016/S0960-9822(00)00728-4 11050388

[B85] Gilley J Coffer PJ Ham J FOXO transcription factors directly activate bim gene expression and promote apoptosis in sympathetic neurons J Cell Biol 2003 162 613 622 10.1083/jcb.200303026 12913110 PMC2173804

[B86] Tang TT Dowbenko D Jackson A Toney L Lewin DA Dent AL Lasky LA The forkhead transcription factor AFX activates apoptosis by induction of the BCL-6 transcriptional repressor J Biol Chem 2002 277 14255 14265 10.1074/jbc.M110901200 11777915

[B87] Boguski MS McCormick F Proteins regulating Ras and its relatives Nature 1993 366 643 654 10.1038/366643a0 8259209

[B88] Bokoch GM Der CJ Emerging concepts in the Ras superfamily of GTP-binding proteins Faseb J 1993 7 750 759 8330683 10.1096/fasebj.7.9.8330683

[B89] Gibbs JB Sigal IS Poe M Scolnick EM Intrinsic GTPase activity distinguishes normal and oncogenic ras p21 molecules Proc Natl Acad Sci USA 1984 81 5704 5708 10.1073/pnas.81.18.5704 6148751 PMC391779

[B90] Shih C Weinberg RA Isolation of a transforming sequence from a human bladder carcinoma cell line Cell 1982 29 161 169 10.1016/0092-8674(82)90100-3 6286138

[B91] Campbell SL Khosravi-Far R Rossman KL Clark GJ Der CJ Increasing complexity of Ras signaling Oncogene 1998 17 1395 1413 10.1038/sj.onc.1202174 9779987

[B92] Schaeffer HJ Weber MJ Mitogen-activated protein kinases: specific messages from ubiquitous messengers Mol Cell Biol 1999 19 2435 2444 10082509 10.1128/mcb.19.4.2435 PMC84036

[B93] Han J Ulevitch RJ Emerging targets for anti-inflammatory therapy Nat Cell Biol 1999 1 E39 40 10.1038/10032 10559893

[B94] Davis RJ Signal transduction by the JNK group of MAP kinases Cell 2000 103 239 252 10.1016/S0092-8674(00)00116-1 11057897

[B95] Chang L Karin M Mammalian MAP kinase signalling cascades Nature 2001 410 37 40 10.1038/35065000 11242034

[B96] Robinson MJ Cobb MH Mitogen-activated protein kinase pathways Curr Opin Cell Biol 1997 9 180 186 10.1016/S0955-0674(97)80061-0 9069255

[B97] Woessmann W Meng YH Mivechi NF An essential role for mitogen-activated protein kinases, ERKs, in preventing heat-induced cell death J Cell Biochem 1999 74 648 662 10.1002/(SICI)1097-4644(19990915)74:4<648::AID-JCB14>3.0.CO;2-6 10440934

[B98] Kyriakis JM Avruch J Sounding the alarm: protein kinase cascades activated by stress and inflammation J Biol Chem 1996 271 24313 24316 10.1074/jbc.271.40.24313 8798679

[B99] Ono K Han J The p38 signal transduction pathway: activation and function Cell Signal 2000 12 1 13 10.1016/S0898-6568(99)00071-6 10676842

[B100] Lubinus M Meier KE Smith EA Gause KC LeRoy EC Trojanowska M Independent effects of platelet-derived growth factor isoforms on mitogen-activated protein kinase activation and mitogenesis in human dermal fibroblasts J Biol Chem 1994 269 9822 9825 7511594

[B101] Shankar S Chen Q Srivastava RK Inhibition of PI3K/AKT and MEK/ERK pathways act synergistically to enhance antiangiogenic effects of EGCG through activation of FOXO transcription factor J Mol Signal 2008 3 7 10.1186/1750-2187-3-7 18355401 PMC2278143

[B102] Bardeesy N DePinho RA Pancreatic cancer biology and genetics Nat Rev Cancer 2002 2 897 909 10.1038/nrc949 12459728

[B103] Li D Xie K Wolff R Abbruzzese JL Pancreatic cancer Lancet 2004 363 1049 1057 10.1016/S0140-6736(04)15841-8 15051286

[B104] Srivastava RK Intracellular mechanisms of TRAIL and its role in cancer therapy Mol Cell Biol Res Commun 2000 4 67 75 10.1006/mcbr.2001.0265 11170835

[B105] Datta SR Brunet A Greenberg ME Cellular survival: a play in three Akts Genes Dev 1999 13 2905 2927 10.1101/gad.13.22.2905 10579998

[B106] Mielke K Herdegen T JNK and p38 stresskinases--degenerative effectors of signal-transduction-cascades in the nervous system Prog Neurobiol 2000 61 45 60 10.1016/S0301-0082(99)00042-8 10759064

[B107] McCubrey JA May WS Duronio V Mufson A Serine/threonine phosphorylation in cytokine signal transduction Leukemia 2000 14 9 21 10.1038/sj.leu.2401657 10637471

[B108] Camps M Nichols A Arkinstall S Dual specificity phosphatases: a gene family for control of MAP kinase function Faseb J 2000 14 6 16 10627275

[B109] Farooq A Zhou MM Structure and regulation of MAPK phosphatases Cell Signal 2004 16 769 779 10.1016/j.cellsig.2003.12.008 15115656

[B110] Hutzen B Willis W Jones S Cen L Deangelis S Fuh B Lin J Dietary agent, benzyl isothiocyanate inhibits signal transducer and activator of transcription 3 phosphorylation and collaborates with sulforaphane in the growth suppression of PANC-1 cancer cells Cancer Cell Int 2009 9 24 10.1186/1475-2867-9-24 19712481 PMC3224892

[B111] Kallifatidis G Rausch V Baumann B Apel A Beckermann BM Groth A Mattern J Li Z Kolb A Moldenhauer G Sulforaphane targets pancreatic tumor-initiating cells by NF-{kappa}B-induced anti-apoptotic signaling Gut 2009 58 949 63 10.1136/gut.2008.149039 18829980

[B112] Pham NA Jacobberger JW Schimmer AD Cao P Gronda M Hedley DW The dietary isothiocyanate sulforaphane targets pathways of apoptosis, cell cycle arrest, and oxidative stress in human pancreatic cancer cells and inhibits tumor growth in severe combined immunodeficient mice Mol Cancer Ther 2004 3 1239 1248 15486191

[B113] Kitamura T Nakae J Kitamura Y Kido Y Biggs WH Wright CV White MF Arden KC Accili D The forkhead transcription factor Foxo1 links insulin signaling to Pdx1 regulation of pancreatic beta cell growth J Clin Invest 2002 110 1839 1847 12488434 10.1172/JCI200216857 PMC151657

[B114] Castrillon DH Miao L Kollipara R Horner JW DePinho RA Suppression of ovarian follicle activation in mice by the transcription factor Foxo3a Science 2003 301 215 218 10.1126/science.1086336 12855809

[B115] Nakae J Biggs WH Kitamura T Cavenee WK Wright CV Arden KC Accili D Regulation of insulin action and pancreatic beta-cell function by mutated alleles of the gene encoding forkhead transcription factor Foxo1 Nat Genet 2002 32 245 253 10.1038/ng890 12219087

[B116] Xia SJ Pressey JG Barr FG Molecular pathogenesis of rhabdomyosarcoma Cancer Biol Ther 2002 1 97 104 12170781 10.4161/cbt.51

[B117] Hu MC Lee DF Xia W Golfman LS Ou-Yang F Yang JY Zou Y Bao S Hanada N Saso H IkappaB kinase promotes tumorigenesis through inhibition of forkhead FOXO3a Cell 2004 117 225 237 10.1016/S0092-8674(04)00302-2 15084260

[B118] Furuyama T Kitayama K Shimoda Y Ogawa M Sone K Yoshida-Araki K Hisatsune H Nishikawa S Nakayama K Nakayama K Abnormal angiogenesis in Foxo1 (Fkhr)-deficient mice J Biol Chem 2004 279 34741 34749 10.1074/jbc.M314214200 15184386

[B119] Dejana E Taddei A Randi AM Foxs and Ets in the transcriptional regulation of endothelial cell differentiation and angiogenesis Biochim Biophys Acta 2007 1775 298 312 17572301 10.1016/j.bbcan.2007.05.003

[B120] Chlench S Mecha Disassa N Hohberg M Hoffmann C Pohlkamp T Beyer G Bongrazio M Da Silva-Azevedo L Baum O Pries AR Zakrzewicz A Regulation of Foxo-1 and the angiopoietin-2/Tie2 system by shear stress FEBS Lett 2007 581 673 680 10.1016/j.febslet.2007.01.028 17258205

[B121] Potente M Urbich C Sasaki K Hofmann WK Heeschen C Aicher A Kollipara R DePinho RA Zeiher AM Dimmeler S Involvement of Foxo transcription factors in angiogenesis and postnatal neovascularization J Clin Invest 2005 115 2382 2392 10.1172/JCI23126 16100571 PMC1184037

[B122] Jin S Shen JN Wang J Huang G Zhou JG Oridonin induced apoptosis through Akt and MAPKs signaling pathways in human osteosarcoma cells Cancer Biol Ther 2007 6 261 268 10.4161/cbt.6.2.3621 17218775

[B123] Schwab TS Madison BB Grauman AR Feldman EL Insulin-like growth factor-I induces the phosphorylation and nuclear exclusion of forkhead transcription factors in human neuroblastoma cells Apoptosis 2005 10 831 840 10.1007/s10495-005-0429-y 16133873

[B124] Srivastava RK Unterman TG Shankar S FOXO transcription factors and VEGF neutralizing antibody enhance antiangiogenic effects of resveratrol Mol Cell Biochem 2010 337 201 212 10.1007/s11010-009-0300-5 20012470 PMC4153854

[B125] Kaneto H Kawamori D Matsuoka TA Kajimoto Y Yamasaki Y Oxidative stress and pancreatic beta-cell dysfunction Am J Ther 2005 12 529 533 10.1097/01.mjt.0000178773.31525.c2 16280646

[B126] Kaneto H Kawamori D Nakatani Y Gorogawa S Matsuoka TA Oxidative stress and the JNK pathway as a potential therapeutic target for diabetes Drug News Perspect 2004 17 447 453 10.1358/dnp.2004.17.7.863704 15514704

[B127] Kawamori D Kaneto H Nakatani Y Matsuoka TA Matsuhisa M Hori M Yamasaki Y The forkhead transcription factor Foxo1 bridges the JNK pathway and the transcription factor PDX-1 through its intracellular translocation J Biol Chem 2006 281 1091 1098 10.1074/jbc.M508510200 16282329

[B128] Stahl M Dijkers PF Kops GJ Lens SM Coffer PJ Burgering BM Medema RH The forkhead transcription factor FoxO regulates transcription of p27Kip1 and Bim in response to IL-2 J Immunol 2002 168 5024 5031 11994454 10.4049/jimmunol.168.10.5024

[B129] Uddin S Hussain AR Siraj AK Manogaran PS Al-Jomah NA Moorji A Atizado V Al-Dayel F Belgaumi A El-Solh H Role of phosphatidylinositol 3'-kinase/AKT pathway in diffuse large B-cell lymphoma survival Blood 2006 108 4178 4186 10.1182/blood-2006-04-016907 16946303

[B130] Daitoku H Fukamizu A FOXO transcription factors in the regulatory networks of longevity J Biochem 2007 141 769 774 10.1093/jb/mvm104 17569704

[B131] van der Heide LP Smidt MP Regulation of FoxO activity by CBP/p300-mediated acetylation Trends Biochem Sci 2005 30 81 86 10.1016/j.tibs.2004.12.002 15691653

[B132] Brunet A Sweeney LB Sturgill JF Chua KF Greer PL Lin Y Tran H Ross SE Mostoslavsky R Cohen HY Stress-dependent regulation of FOXO transcription factors by the SIRT1 deacetylase Science 2004 303 2011 2015 10.1126/science.1094637 14976264

[B133] Imai S Armstrong CM Kaeberlein M Guarente L Transcriptional silencing and longevity protein Sir2 is an NAD-dependent histone deacetylase Nature 2000 403 795 800 10.1038/35001622 10693811

[B134] Shankar S Ganapathy S Hingorani SR Srivastava RK EGCG inhibits growth, invasion, angiogenesis and metastasis of pancreatic cancer Front Biosci 2008 13 440 452 10.2741/2691 17981559

[B135] Shankar S Siddiqui I Srivastava RK Molecular mechanisms of resveratrol (3,4,5-trihydroxy-trans-stilbene) and its interaction with TNF-related apoptosis inducing ligand (TRAIL) in androgen-insensitive prostate cancer cells Mol Cell Biochem 2007 304 273 285 10.1007/s11010-007-9510-x 17636462

